# Male-specific roles of lincRNA in *C. elegans* fertility

**DOI:** 10.3389/fcell.2023.1115605

**Published:** 2023-03-23

**Authors:** Reut Shabtai, Yonatan B. Tzur

**Affiliations:** Department of Genetics, Institute of Life Sciences, The Hebrew University of Jerusalem, Jerusalem, Israel

**Keywords:** lncRNA, lincRNA, long intergenic non-coding RNA, spermatogenesis, fertility, *C. elegans*

## Abstract

The testis is the mammalian tissue with the highest expression levels of long intergenic non-coding RNAs (lincRNAs). However, most *in vivo* models have not found significant reductions in male fertility when highly expressed lincRNA genes were removed. This suggests that certain lincRNAs may act redundantly or lack functional roles. In the genome of the nematode *Caenorhabditis elegans,* there is an order of magnitude fewer lincRNA genes than in mammals. This characteristic lowers the potential for redundancy, making it an ideal model to test these possibilities. We identified five highly and dynamically expressed lincRNAs in male *C. elegans* gonads and quantified the fertility of worm strains in which these genes were removed. In contrast to the hermaphrodites of deletion strains, which exhibited no significant reductions in broods, smaller brood sizes were observed in the progeny of males of three of the lincRNA deleted strains. This demonstrates reduced male fertility in worms with those genes removed. Interestingly, reduced brood size was statistically significant only in the last days of egg laying in two of these strains. This suggests the effect is due to early deterioration and aging of the transferred sperm. We detected a mild increase in embryonic lethality in only one of the strains, supporting the possibility that these lincRNAs do not affect fertility through critical roles in essential meiotic processes. Together our results indicate a sexually dimorphic outcome on fertility when lincRNA are removed and show that, unlike mammals, individual lincRNAs in *C. elegans* do play significant roles in male fertility.

## Introduction

Spermatogenesis is a complex developmental plan in which germ stem cells differentiate into mature spermatozoa. Spermatogenesis consists of several stages including primordial germ cell expansion, two meiotic divisions, and differentiation into spermatozoa (L'Hernault, 1997; Yoshida, 2010; Chu and Shakes, 2013; Ellis and Stanfield, 2014; Griswold, 2016). During the meiotic stage, the number of chromosomes is reduced by half to create haploid cells. This is achieved by unique chromosome interactions, including homologous chromosome pairing, synapsis, and recombination (Colaiacovo et al., 2003; Couteau and Zetka, 2005; Hayashi et al., 2010; Schild-Prufert et al., 2011; Reichman et al., 2017). Unlike many other stages in spermatogenesis, meiosis is also executed in a similar fashion in oogenesis. Failure to complete meiotic specific processes, in many cases, leads to apoptotic programmed cell death (Ye et al., 2014).

Long non-coding RNAs (lncRNAs) are transcribed in a similar process as mRNAs, and are often capped, spliced, and poly-adenylated, but not translated. LncRNAs are transcribed from tens of thousands of loci in the human genome (reviewed in (Ulitsky and Bartel, 2013; Fatica and Bozzoni, 2014; Marques and Ponting, 2014; Blythe et al., 2016; Melissari and Grote, 2016; Deniz and Erman, 2017; Kopp and Mendell, 2018; Fico et al., 2019; Shields et al., 2019). Approximately half of the human lncRNAs are transcribed from genomic loci that don’t overlap with coding genes and are denoted as long intergenic non-coding RNAs (lincRNAs) (Uszczynska-Ratajczak et al., 2018).

Testis has the most complex transcriptome and expresses the highest levels and the largest repertoire of lncRNAs compared with all other mammalian tissues (Soumillon et al., 2013; Necsulea et al., 2014; Washietl et al., 2014; Hezroni et al., 2015; Hong et al., 2018). Surprisingly, in almost all cases, lncRNA *in vivo* knockout or knockdown mammalian models failed to exhibit significant reductions in male fertility (*e.g.*, (Mehta et al., 2021; Chadourne et al., 2021; Li et al., 2021; Zhang et al., 2021). Several hypotheses were suggested to explain this conundrum, including promiscuous transcription due to rapid changes in chromatin structure and functional redundancy of several lncRNAs (Reviewed in (Tzur, 2022).

Wild-type *C. elegans* nematodes exist as hermaphrodites and males. Hermaphrodites, which have two X chromosomes, produce sperm during larval stages, but switch to oogenesis in adulthood (L'Hernault, 1997; Chu and Shakes, 2013; Ellis and Stanfield, 2014; Griswold, 2016). Oocytes can be fertilized by self-sperm or by sperm transferred by males. Several genetic mutations lead to hermaphrodites that cannot self-fertilize, making them functionally females. Male *C. elegans* worms have only one X chromosome, produce only sperm, and are present at ∼0.1% of the population under normal laboratory conditions. In both male and hermaphrodite gonads, the germ cells are arranged in a spatio-temporal manner from proliferative stem cells to mature gametes (L'Hernault, 1997; Chu and Shakes, 2013; Ellis and Stanfield, 2014; Griswold, 2016). Humans and *C. elegans* have a similar number of coding genes, yet only a few hundred lincRNA genes were found in the latter’s genome (Nam and Bartel, 2012; Akay et al., 2019). This order of magnitude fewer lincRNA genes reduces the likelihood of redundancy in the lincRNA roles present during the worm’s spermatogenesis process.

The study of fertility and meiosis in *C. elegans* has uncovered many evolutionary conserved processes (reviewed in (Hubbard and Greenstein, 2005; Lui and Colaiacovo, 2013; Hillers et al., 2017; Hubbard and Schedl, 2019). However, most of this work has examined hermaphrodites. Far less is known about fertility in males [reviewed in (LHernault, 2009; Ellis and Stanfield, 2014)]. Similar to hermaphrodites, the distal side of the male gonad cells undergo proliferation and complete meiotic reductional division as they move proximally. This step includes pairing, synapsis and crossovers of homologous chromosomes (Chu and Shakes, 2013). Similar to other metazoans, in *C. elegans* males the meiotic divisions create four gametes (in contrast to oogenesis in which only one oocyte is formed from each progenitor cell), as well as shedding most of the cytoplasmic components and formation of a residual body (Shakes et al., 2009; Chu and Shakes, 2013) and chromatin compaction assisted by protamine proteins as spermatids develop into mature spermatozoa (Nishimura and LHernault, 2017).

We have previously analyzed the transcriptomic changes along the stages of oogenesis and spermatogenesis in *C. elegans.* We used laser capture microdissection to cut both hermaphrodite and male gonads into 10 sequential segments (Tzur et al., 2018). RNASeq analysis allowed us to quantitatively compare gene expression between the two gametogenesis processes and between different stages within oogenesis or spermatogenesis. To assess the roles of lincRNAs in oogenesis, we previously used these databases and found lincRNAs that are highly and dynamically expressed in the hermaphrodite gonad (Ishtayeh et al., 2021). We engineered full genomic homozygous deletion strains for these lincRNA genes, thus preventing expression of any part of the gene ((Ishtayeh et al., 2021) and Methods). Surprisingly, we found no change in hermaphrodite fertility without those lincRNAs (Ishtayeh et al., 2021). These worms also did not have higher than wild-type rates of embryonic lethality, germline apoptosis, defects in synapsis of homologous chromosomes, or bivalent structure of mature oocytes. Therefore, these lincRNAs are not required for normal spermatogenesis and oogenesis in hermaphrodites (Ishtayeh et al., 2021). However, the effect of these lincRNA gene deletions on male fertility has not been explored.

In this article we report our analysis of male fertility in worm strains in which we deleted the five lincRNA genes which we previously determined are redundant for hermaphrodite fertility (Ishtayeh et al., 2021). In three of those strains, we found a significant reduction in the brood size of mutant males, and in one we found a mild increase in embryonic lethality. This stands in contrast with hermaphrodites, despite the fact that they also produce sperm. Thus, our work indicates that some of the lincRNAs in worms are required specifically for male fertility.

## Materials and methods

### Strains and alleles

The *fem-2* worms were cultured at 15°C and transferred to 25°C prior to progeny quantification experiments (see below). All other strains were cultured under standard conditions at 20°C (Brenner, 1974). The N2 Bristol strain was utilized as the wild-type background. Worms were grown on NGM plates with *Escherichia coli* OP50 (Brenner, 1974).

The following mutations were used in this study: LGI: *linc-9(huj24),* LGII: *linc-4(huj25),* LGIII: *fem-2(b245),* LGV: *linc-168(huj10),* LGX: *linc-7(huj9), linc-20(huj21).*

Engineering the lincRNA deletion strains was reported in (Ishtayeh et al., 2021). In short, we used the CRISPR genome editing method described in (Achache et al., 2019) with gRNAs directed to regions upstream and downstream of the gene. This strategy ensures that every single base of the gene is removed from the genome. Deletions were identified by PCR and complete deletion of the genes was verified by Sanger sequencing. Sequences and molecular data are detailed in Supplementary Table S1 in (Ishtayeh et al., 2021). After five times outcrossing, to minimize possible off-target mutations, we established homozygous strains which were used in this study.

### LincRNA selection

To find lincRNAs which affect male but not hermaphrodite fertility we used lincRNAs reported in (Ishtayeh et al., 2021), in which full lincRNA deletion alleles were engineered and five times outcrossed strains were carefully tested for hermaphrodite’s fertility related phenotypes. The strains that showed no effect were selected and the level of the transcripts along the male gonad were evaluated from the datasets published in (Tzur et al., 2018). LincRNAs with dynamic expression and at least an average of six counts along the ten segments were selected for analysis in males.

### RNA level analysis

RNASeq data of the lincRNAs and meiotic genes presented in Supplementary Figure S1 were extracted from the male analyses reported in (Tzur et al., 2018). Normalized values of the ten sequential segments from two male gonads were averaged, increased by one and log_2_ transformed. The resulting values were plotted as data points of the values vs. the 10 segments from (1) proliferating to the (10) spermatozoa stage.

### Progeny quantification

To quantify the progeny, we used the well based method of Plate Phenotype (Fay, 2013). Worms were feminized by transferring *fem-2(b245)* L4 larvae from 15°C to 25°C. 48–72 h after the temperature shift, L4 *fem-2(b245)* F1 progeny were individually isolated to plates with five young adult males of the appropriate genotype (wild type or with specific lincRNA deletion) and maintained at 20°C until the end of the experiment. After 24 h, the female and male worms were transferred to a new plate. The female was transferred four additional times every 24 h.

The embryos and hatched progeny in each plate were counted at the time of the adult transfer and 24 h later. Only data from worms that survived until completion of the experiment were used. The n value was: *linc-4* = 19*, linc-7* = 17*, linc-9* = 16*, linc-20* = 11, *linc-168* = 16, and wild type = 64 individual mated females. To verify full feminization, at least three plates with individual *fem-2(b245)* worms were isolated without males and monitored for zero F1 progeny. Mating feminized worms with wild-type males served as control for the effects of males with lincRNA deletion.

### Embryonic lethality quantification

Embryonic lethality was quantified as in (Achache et al., 2019). In short, the number of larvae and embryos were counted in each plate and used for progeny quantification (see above) at two time points: After the transfer of the P0 individuals, and 24 h later. *C. elegans* L1 larvae hatch ∼800 min after fertilization (Sulston et al., 1983), therefore any embryo found on the plate during the second count (24 h after removal of the P0 worms) was termed “dead”. For each mated feminized worm, the total number of dead embryos was divided by the total progeny (larvae and dead embryos) to determine this worm’s progeny embryo lethality.

### Statistics

For the data presented in Figure 4B we used the Fisher’s exact test. For all other analyses we used the two tailed Mann-Whitney U test.

## Results

### Analysis of lincRNA gene expression

Germ cells in the gonad of both male and hermaphrodite worms are arranged in spatiotemporal pattern from proliferative cells to mature gametes (L'Hernault, 1997; Chu and Shakes, 2013; Ellis and Stanfield, 2014; Griswold, 2016). We have utilized this setup in the past to determine the transcriptome of 10 sequential segments in along gonads of hermaphrodites and males to analyze gene expression during oogenesis and spermatogenesis respectively (Tzur et al., 2018). In that work, RNASeq analysis was used to quantify the transcriptome in 10 sequential segments of gonads from both sexes (Tzur et al., 2018). In a follow-up work we used the datasets to identify lincRNAs genes that are highly and dynamically expressed in the hermaphrodite gonads (Ishtayeh et al., 2021). Using CRISPR genome engineering we created homozygote worm strains with a full deletion of one of these lincRNAs. Hermaphrodite worms of these strains showed no change in fertility under normal laboratory conditions (Ishtayeh et al., 2021). The lack of fertility reduction in these strains indicates the lincRNAs are redundant for both oogenesis and spermatogenesis in hermaphrodites. Previous reports identified genes that play a role in male, but not hermaphrodite, spermatogenesis [*e.g.*, (Stanfield and Villeneuve, 2006)]. To find lincRNA genes with fertility roles only in males we focused on lincRNAs found to be redundant for hermaphrodite fertility (Ishtayeh et al., 2021) and have significant and dynamic expression in the male gonad (see Methods).

**FIGURE 1:**
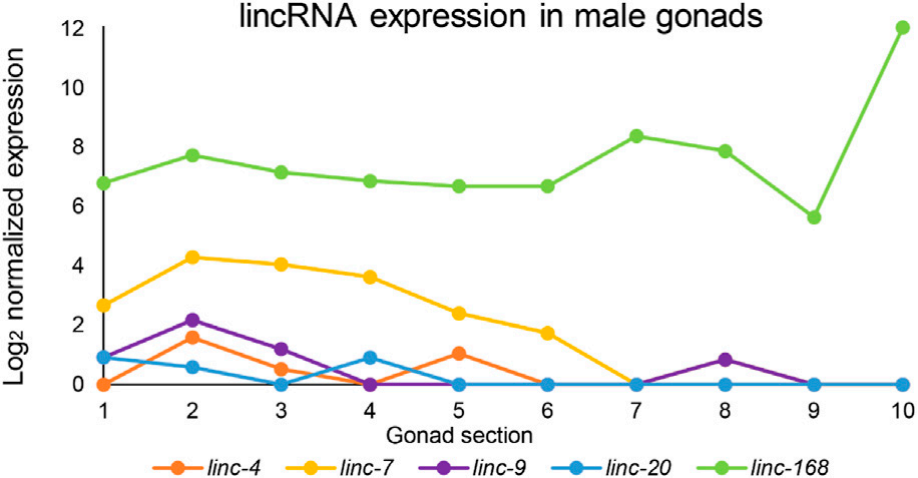
Expression patterns of lincRNAs in the male gonad. Log_2_ of normalized expression values of five lincRNAs with high levels of expression along the male gonad from proliferative to mature sperm stages. *X*-axis numbers correspond to the segments used for the analysis and refer to the following stages: 1-2 proliferative, 2–4 leptotene/zygotene, 5-6 pachytene, 7-8 condensation, and division, 9-10 spermiogenesis. Adapted from (Tzur et al., 2018).

We used the RNAseq datasets of male gonad sections published in (Tzur et al., 2018) to find the expression patterns of these lincRNAs in males. We found that five lincRNAs (*linc-4, linc-7, linc-9, linc-20*, and *linc168*) which were found to be redundant for hermaphrodite fertility (Ishtayeh et al., 2021) are highly and dynamically expressed in males (Figure 1). Two of these lincRNAs, *linc-7* and *linc-168,* are among the highest expressed transcripts in the male gonad. The peak expression of *linc-7* and *linc-168* is higher than many genes known to be essential for successful spermatogenesis (*e.g., mpk-1, syp-1*, and *htp-3*; Supplementary Figure S1). Only the male-specific protein gene *msp-51* has a peak expression higher than *linc-168*. In contrast, most lincRNAs are generally expressed at lower levels than coding genes in both mammals and *C. elegans* (Nam and Bartel, 2012; Soumillon et al., 2013; Akay et al., 2019).

Our analysis indicates several similarities between oogenesis and spermatogenesis lincRNA expression. *Ilinc-7* and *linc-168* are the lincRNAs with the highest expression in both developmental processes [Figure 1 and (Ishtayeh et al., 2021)]. Additionally, *linc-7* expression is higher at the first half of the gonad in both sexes, while *linc-168* expression is mostly stable [Figure 1 and (Ishtayeh et al., 2021)]. *linc-7* and *linc-20,* which are both transcribed from the X chromosome, are expressed during the early parts of the gonads [Figure 1 and (Ishtayeh et al., 2021)]. This stands in contrast to most X-linked genes which are silenced during this stage in both males and hermaphrodites (Kelly et al., 2002; Bean et al., 2004; Tzur et al., 2018). Some differences do exist in the expression pattern of these lincRNAs between spermatogenesis and oogenesis. For example, *linc-9* is expressed at the highest levels during late pachytene and diplotene during oogenesis (Ishtayeh et al., 2021), but is primarily, present in premeiotic and leptotene/zygotene stages in spermatogenesis (Figure 1). We conclude that in male gonads, expression patterns of the lincRNAs discussed here are mostly high and share similar dynamics to their expression in hermaphrodite gonads.

### Deletion of *linc-7, linc-9, and linc-168* leads to reduced male fertility

**FIGURE 2:**
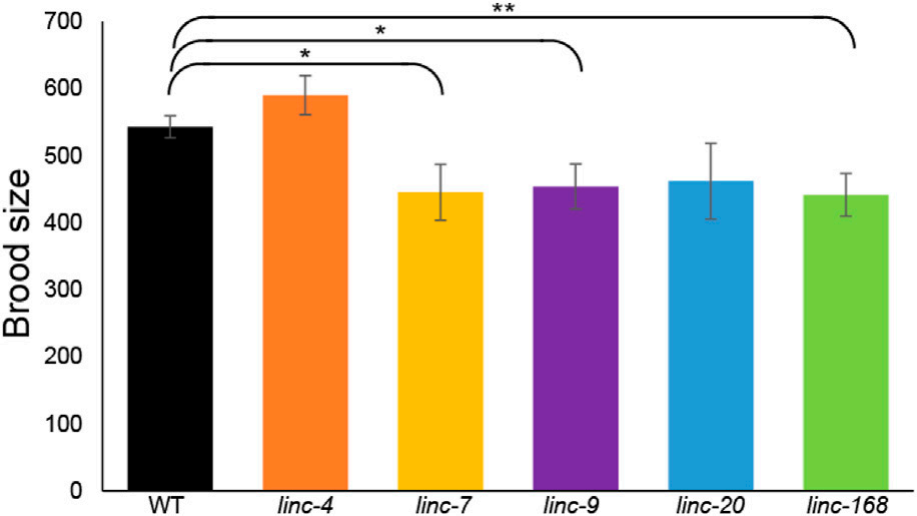
Deletion of three lincRNA genes leads to reduced male fertility. Average progeny brood sizes for females mated with males of the indicated lines. Mann-Whitney *p-*value: * <0.05, ** <0.01.

To test male fertility without the interference of hermaphrodite self-fertilization we used *fem-2(b245)* worms which contain a temperature-sensitive mutation that causes XX worms to be functionally females at 25°C [see Methods (Hodgkin, 1986)]. We isolated L4 feminized larvae together with males for 2 days, removed the males, and continued to score the progeny for four more days (see Methods). Fertilized females mated with wild-type males laid an average of 543 ± 17 embryos during this 6 days period, in line with previous reports of mated wild-type females (Pickett et al., 2013). In contrast, females mated with male worms with lincRNA deletions showed a significant reduction in the number of laid embryos in three of the five strains tested (Figure 2; *linc-7, linc-9, and linc-168*, *p*-value by the Mann-Whitney test = 0.04, 0.02, and 0.008 respectively). We observed a similar, but not statistically significant, reduction for *linc-20* males (Figure 2). Interestingly, *linc-9*, and *linc-20* are highly paralogous (Ishtayeh et al., 2021), suggesting they may be partially redundant [albeit see (Rappaport et al., 2022)]. We note that *linc-4* males had brood size larger than those of the wild-type males, however, this was not statistically significant (*p-*value >0.1 by the Mann-Whitney test). In conclusion three out of the five lincRNA deletion strains have a male-specific reduction in fertility.

### No substantial increase in embryonic lethality of the progeny of lincRNAs deletion mutants

**FIGURE 3:**
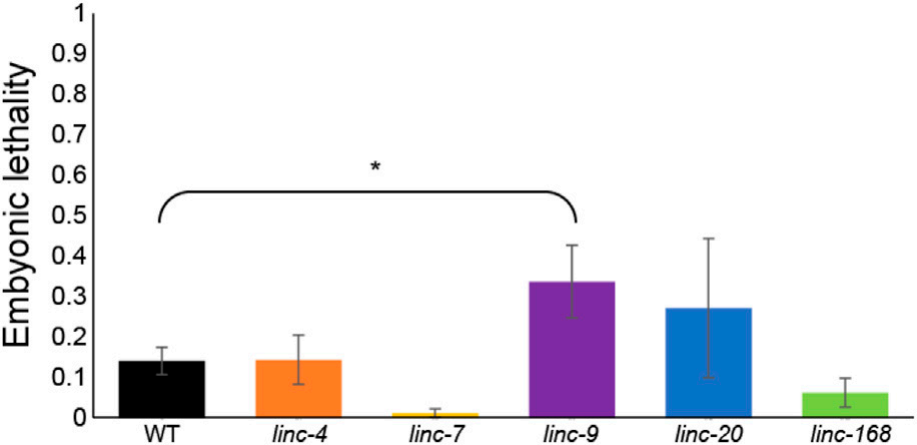
Progeny of males with deletions in lincRNA genes do not undergo substantial embryonic lethality. Average embryonic lethality of the progeny of males of the indicated genotypes. Mann-Whitney *p-*value: * <0.05.

Several mutations that create aberrations in gametogenesis, especially those connected with meiotic processes, were shown to lead to embryonic lethality of the progeny. For example, mutations that hamper homologous chromosome pairing (Phillips and Dernburg, 2006), synapsis (MacQueen et al., 2002; Smolikov et al., 2007a; Smolikov et al., 2007b) and recombination, prevent correct transfer of chromosomes to the egg and sperm. This prevents successful completion of embryogenesis of the progeny. During oogenesis, many meiocytes that fail to correctly undergo synapsis and recombination are removed by apoptosis and do not end up as oocytes. In the worm’s male germline, these failures don’t lead to apoptosis, meaning even mild defects can increase embryonic lethality (Bhalla and Dernburg, 2005; Jaramillo-Lambert et al., 2010; Ye et al., 2014; Bohr et al., 2016). To find if the reduced male fertility we found in three of the lincRNA deletions (Figure 2) also lead to increased embryonic lethality we quantified the number of hatched and unhatched embryos. In the progeny of the lincRNA mutant males, we did not observe any substantial increase in embryonic lethality (Figure 3). Notably *linc-9* and *linc-20* did show a slight increase in embryonic lethality (0.4% vs. 0.1% compared to wild-type males), but only *linc-9* showed a statistically significant increase. These results raise the possibility that these two extremely paralogous lincRNAs may play redundant roles. Taken together these mutations do not lead to any relevant increase in embryonic lethality, suggesting the reduction in fertility isn’t caused by meiotic failures.

### Most of the reduction in fertility in females mated with *linc-7* and *linc-168* males occurs during late stages of egg laying.

**FIGURE 4:**
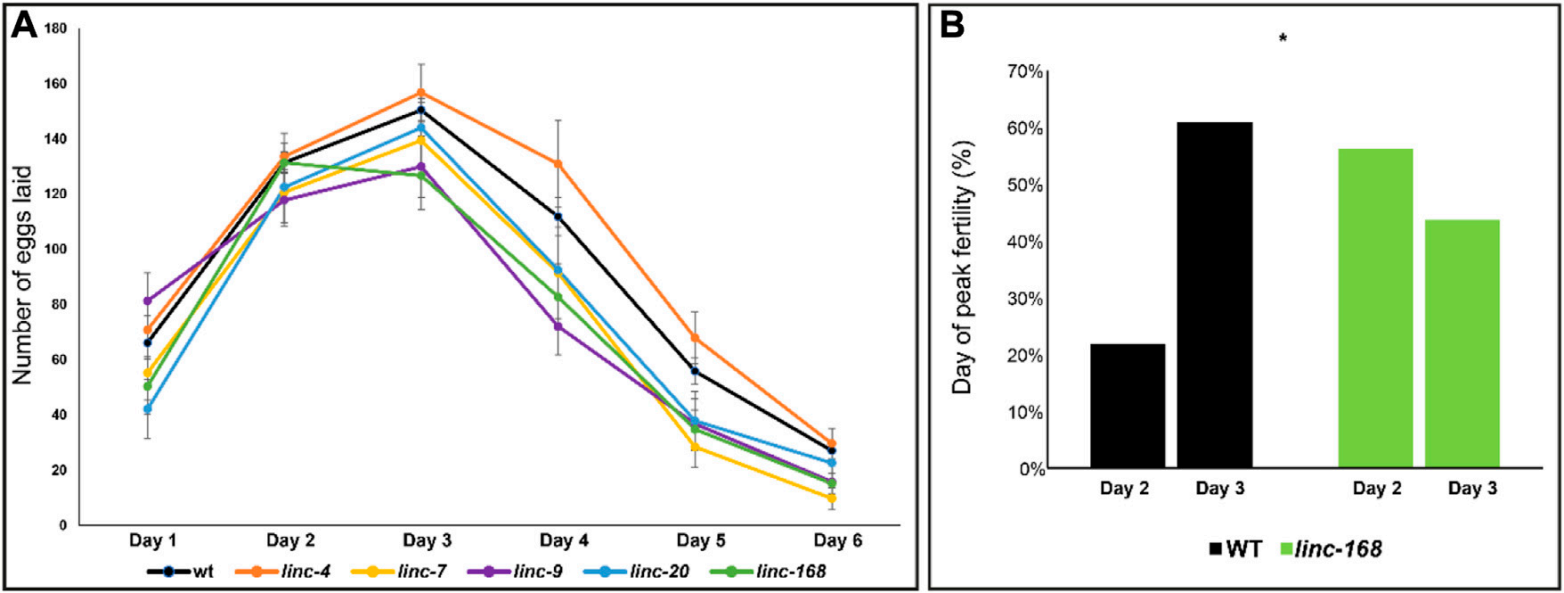
Dynamics of brood size along the reproductive term of lincRNA deleted males. **(A)** Average progeny brood size for females mated with males of the indicated genotypes along six 24 h intervals. *X*-axis timepoints refer to days post fertilization. **(B)** Percentage of embryos laid on day 2 and day 3 by progeny of WT and *linc-168* males.

The egg-laying dynamics in *C. elegans* change under different conditions. For example, hermaphrodite worms reach their egg-laying peak during their second day of adulthood while fertilized females reach it on the third day [Figure 4 and (Pickett et al., 2013)]. Moreover, several mutations were shown to change the dynamics of egg-laying [e.g., (Kadandale and Singson, 2004)].

To find whether the deletion of the lincRNAs resulted in a change in the egg-laying dynamics we compared the brood size in each of the 24 h intervals over the 6 days period (Figure 4A). We found that the general dynamics of egg-laying of females mated with the lincRNA mutant males were similar to wild-type males, and most showed a peak of brood production in the third day of adulthood (Figure 4A). The only exception was *linc-168* in which 56% of the mated worms reached a peak on the second day of adulthood compared to 24% of the worms mated with wild-type males (Figure 4B; *p*-value <0.05 by the Fisher exact test). In the three strains that demonstrated reduced fertility we found variation in the time points in which most of the reduction compared to wild type was measured. *linc-9* displayed a non-significant increase in brood size the first day after mating but had significant reduction in the number of progeny compared to wild-type in days that followed (Figure 4A; *p*-value <0.05 by the Mann-Whitney test). Conversely, in *linc-7* and *linc-168*, we found reduced brood size across the entire tested period, but we only found a statistically significant reduction in broods on days five and six after mating. Given that the total brood size of *linc-9* is larger than *linc-7* and *linc-168* males, these results raise the possibility for a more substantial effect in the last days of egg laying after fertilization by *linc-7* and *linc-168*. We conclude that the reduction in brood size of females mated by lincRNA deletion strain males occurs at different times along the fertility term depending on the removed gene.

## Discussion

### How can the same mutation lead to male-specific fertility phenotype in a process that occurs in both sexes?

Sexual dimorphism manifests as different phenotypes between males and females with a similar genetic background. In *C. elegans* it is possible to test this phenomenon for spermatogenesis since both sexes produce sperm. Stanfield and Villeneuve reported in the past that a mutation in *swm-1* reduces sperm activation, and therefore fertility in male worms but not in hermaphrodites (Stanfield and Villeneuve, 2006). Conversely, mutations in genes from the *spe-8* group are required for hermaphrodite self-fertility, but males are fully fertile (LHernault et al., 1988; Shakes and Ward, 1989). Mutant lincRNA genes tested in this work displayed effects on male fertility but did not change reproduction of hermaphrodites. Despite the fact that fertility in both sexes depends on sperm, surprisingly, we found a significant reduction in male fertility in three out of the five mutants tested.

When comparing the effects of these lincRNAs in hermaphrodites [as reported in (Ishtayeh et al., 2021)] to their effects in males we report here, several similarities exist. In both sexes two of these lincRNAs (*linc-4* and *linc-20*) are redundant for fertility. Also, deletion of none of these lincRNA led to high embryonic lethality. On the other hand, here we show that three lincRNAs are required for normal fertility in males but not in hermaphrodites, although both sexes produce sperm.

What could lead to the difference between male and hermaphrodite when it comes to lack of lincRNA genes? Several hypotheses can be envisioned. Spermatozoa differs in males and hermaphrodites. For example, mature sperm in males are larger and faster than those produced by hermaphrodites (LaMunyon and Ward, 1998). Moreover, unlike hermaphrodites, male sperm must crawl from the vulva to the spermatheca. Therefore, subtle changes in crawling efficiency or orientation will be more critical for successful fertilization in male vs. hermaphrodite sperm. It is thus possible that the lincRNA mutations affect specific features in male sperm such as size or activation. Second, although the basic genetic program of spermatogenesis is similar between males and hermaphrodites, some variations in gene expression are present (L'Hernault, 1997; Ellis and Stanfield, 2014). Moreover, hermaphrodites have two X chromosomes whereas males have only one. Of note, two of the lincRNAs, *linc-7*, and *linc-20,* are coded on the X chromosome, and contrary to most X-linked genes they are expressed in male gonads and early stages of hermaphrodite oogenesis [Figure 1 and (Tzur et al., 2018; Ishtayeh et al., 2021)]. lincRNA deletion can affect gene expression within the worm’s germline [e.g., (Ishtayeh et al., 2021)], potentially leading to effects on male-specific pathways. Third, fertility could be reduced due to effects on the soma and not the germline. This could be manifested by specific sex-related roles of the soma, such as the requirement for males to interreact and transfer the sperm to the hermaphrodite, or specific interactions between somatic and germ cells within the same organism. Deletion of the lincRNA could result in a male-specific somatic change that will affect sperm quality and or transfer efficiency.

### The reduced fertility of *linc-7* and *linc-168* is probably due to reduced quality or early aging of the transferred sperm.

Given these options, what could be the cause of the reduced fertility in the specific strains described here? Failure in chromosome segregation, whether it be meiotic or mitotic, leads to embryonic lethality. In hermaphrodites, but not in males, these are also accompanied by apoptosis. Therefore, meiotic failures are expected to lead to higher rates of embryonic lethality of the progeny if they occur in males. The only strain in which statistically significant embryonic lethality of the progeny is observed is *linc-9*. Yet, even in that case, the increase was extremely mild, below 1%, far less than the reduction in fertility. This makes the possibility of meiotic critical errors less likely to be the cause of the reduced fertility in this strain.

The analysis of brood size dynamics in the days following the mating is more informative. In our experiments the feminized worms were mated as L4 larvae for 48 h. The sperm which was transferred to them by the males had to stay functional for the entire period of the experiment (∼6 days). Consistent lower progeny (as compared to mating with wild type males) throughout the experiment period would suggest an overall lower sperm quality, whereas reduction only in the last days of the experiment would suggest that the sperm that was transferred deteriorated within the mated females. The significant reduction in brood from the second to the sixth day after mating in *linc-9,* correspond to the first scenario and suggests that it is lower quality sperm that leads to the reduced brood sizes. This lower quality could result in a reduced number of successful fertilization events already on the second day. On the other hand, the specific reduction in brood size of females mated with *linc-7* and *linc-168* during days five and six can correspond to the second option, and therefore the sperm that was transferred “aged” and deteriorated after a few days. It is also possible that the number of sperm transferred was lower. However, the fact that we introduced an excess number of males per female (see methods) suggests the former option is more likely. These results raise the question regarding the molecular mechanism by which *linc-7, linc-9*, and *linc-168* promote normal male fertility*.* Very little is known about the molecular roles of lincRNAs in *C. elegans*. We showed that deletion of *linc-4* leads to transcriptomic changes, with a significant enrichment of germline genes and genes involved in cuticle formation (Ishtayeh et al., 2021). It is therefore possible that the three lincRNAs that have roles in male fertility also work *via* transcription modulation. Additionally, the very high levels of *linc-168* and *linc-7* raise the possibility they act as competitive inhibitors for RNA, proteins and specific genomic sites, as was previously demonstrated for lincRNAs in mammals (Ulitsky and Bartel, 2013; Fatica and Bozzoni, 2014; Marques and Ponting, 2014; Blythe et al., 2016; Melissari and Grote, 2016; Deniz and Erman, 2017; Kopp and Mendell, 2018; Fico et al., 2019; Shields et al., 2019).

### Is it possible that in invertebrates there is less lncRNA redundancy than in mammals?

LincRNAs have generally low sequence evolutionary conservation in both mammals and nematodes (Ulitsky and Bartel, 2013; Fatica and Bozzoni, 2014; Marques and Ponting, 2014; Blythe et al., 2016; Melissari and Grote, 2016; Deniz and Erman, 2017; Kopp and Mendell, 2018; Fico et al., 2019; Shields et al., 2019). Previous works suggested that lincRNAs with more prominent physiological roles will also show higher sequence conservation. However, *linc-7, linc-9* and *linc-168* have very low conservation even when compared to *C. brenneri, C. briggsae*, and *C. remanei* (Blast score lower than 45).

Our results stand in contrast to mice models in which many knock-outs of a single lincRNAs didn’t significantly reduce male fertility. We propose that similar to *Drosophila*, in which single mutations in many lncRNAs genes reduced male fertility (Wen et al., 2016), *C. elegans* highly expressed lincRNAs aren’t redundant. However, our results did raise the possibility that some level of redundancy may be present between *linc-9* and *linc-20.* It will be interesting to revisit the roles of lncRNAs in mammalian spermatogenesis and find groups of lncRNAs that perform the same molecular role. Taken together we conclude that the reduced fertility in males could be the result of either the number, quality, or longevity of the sperm. Future studies will probably test these models and find how the lincRNAs affect mating, the number, and the quality of the transferred sperm.

## Data Availability

The original contributions presented in the study are included in the article/Supplementary Material, further inquiries can be directed to the corresponding author.

## Supplementary material

Supplementary Figure S1 Comparison between the expression patterns of *linc-7, linc-168*, and prominent meiotic genes. Log_2_ of normalized expression values of *linc-7 and linc-168* and the indicated meiotic genes along the male gonad from proliferative (1) to mature sperm (10) stages. X-axis numbers correspond to the segments used for the analysis and refer to the following stages: 1-2 proliferative, 2-4 leptotene/zygotene, 5-6 pachytene, 7-8 condensation and division, 9-10 spermiogenesis. Note the maximum levels of the lincRNAs are higher than those of the meiotic genes. Adapted from (Tzur et al., 2018).
