# Supplementary figures and images for: Male-specific roles of lincRNA in C. elegans fertility
Source: Front Cell Dev Biol. 2023 Mar 23;11:1115605. doi: 10.3389/fcell.2023.1115605 (PMC10076526; doi:10.3389/fcell.2023.1115605)

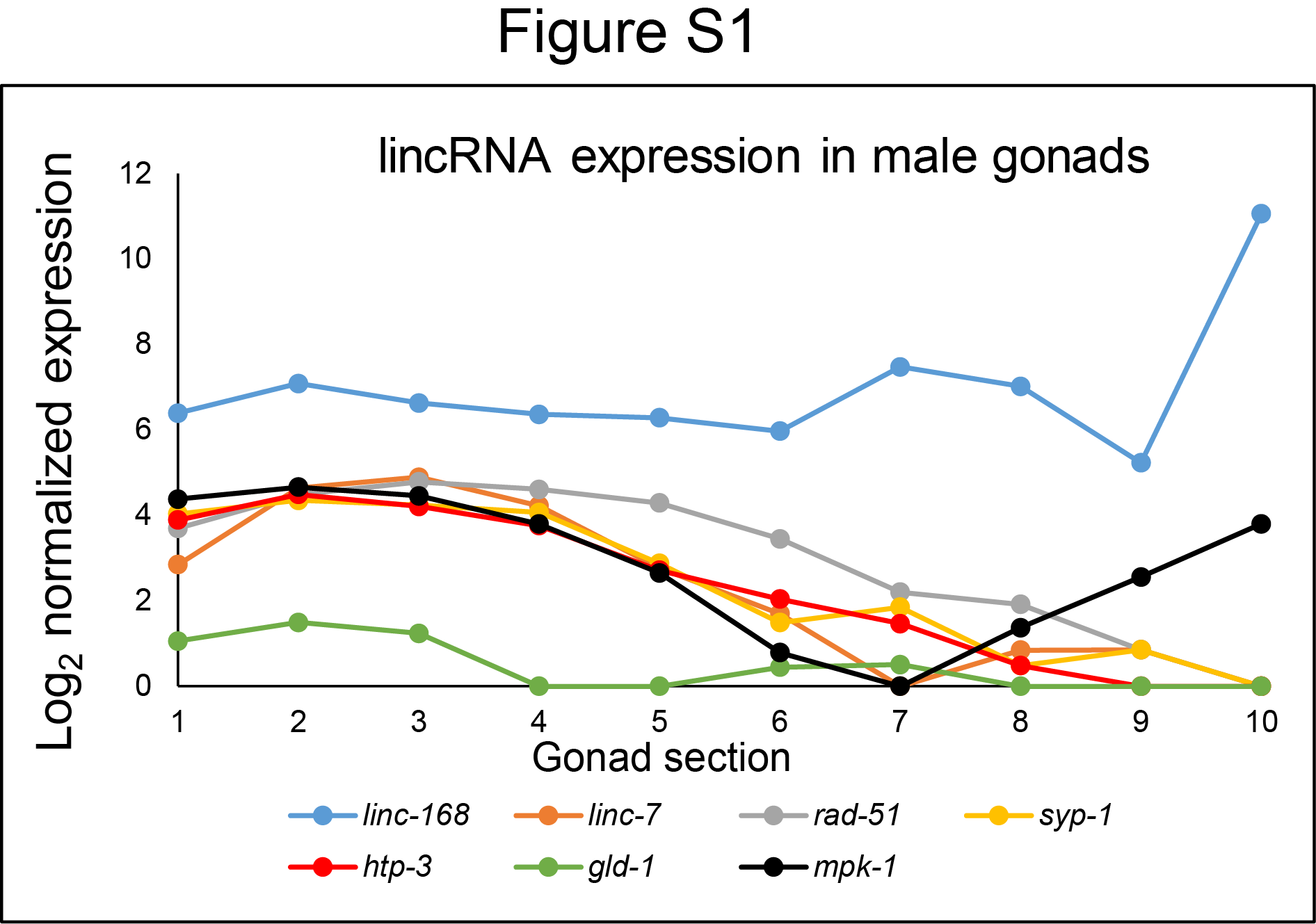

Supplement: Supplementary file 1 [file Image1.TIF]
